# Universal screening for latent and active tuberculosis (TB) in asylum seeking children, Bochum and Hamburg, Germany, September 2015 to November 2016

**DOI:** 10.2807/1560-7917.ES.2018.23.12.17-00536

**Published:** 2018-03-22

**Authors:** Maya Mueller-Hermelink, Robin Kobbe, Benedikt Methling, Cornelius Rau, Ulf Schulze-Sturm, Isa Auer, Frank Ahrens, Folke Brinkmann

**Affiliations:** 1 Altona Children’s Hospital, Department of Pulmonology, Hamburg, Germany; 2 These authors contributed equally to this article; 3 University Medical Centre Hamburg-Eppendorf, Department of Pediatrics, Hamburg, Germany; 4 University Children`s Hospital Bochum- Germany, Department of Pulmonology, Bochum, Germany; 5 German Red Cross, Chapter Hamburg-Harburg, Hamburg, Germany; 6 Center of Applied Sciences of Health (CASH), Leuphana University, Lueneburg, Germany

**Keywords:** Tuberculosis, surveillance, public health policy, refugees, children

## Abstract

In Germany, the incidence of tuberculosis (TB) in children has been on the rise since 2009. High numbers of foreign-born asylum seekers have contributed considerably to the disease burden. Therefore, effective screening strategies for latent TB infection (LTBI) and active TB in asylum seeking children are needed. **Aim:** Our aim was to investigate the prevalence of LTBI and active TB in asylum seeking children up to 15 years of age in two geographic regions in Germany. **Methods:** Screening for TB was performed in children in asylum seeker reception centres by tuberculin skin test (TST) or interferon gamma release assay (IGRA). Children with positive results were evaluated for active TB. Additionally, country of origin, sex, travel time, TB symptoms, TB contact and Bacille Calmette-Guérin (BCG) vaccination status were registered. **Results:** Of 968 screened children 66 (6.8%) had TB infection (58 LTBI, 8 active TB). LTBI prevalence was similar in children from high (Afghanistan) and low (Syria) incidence countries (8.7% vs 6.4%). There were no differences regarding sex, age or travel time between infected and non-infected children. Children under the age of 6 years were at higher risk of progression to active TB (19% vs 2% respectively, p=0,07). Most children (7/8) with active TB were asymptomatic at the time of diagnosis. None of the children had been knowingly exposed to TB. **Conclusions:** Asylum seeking children from high and low incidence countries are both at risk of developing LTBI or active TB. Universal TB screening for all asylum seeking children should be considered.

## Introduction

In recent years, Germany experienced a major increase in the number of migrants. Of 890,000 refugees/asylum seekers (hereafter referred to as asylum seekers) who reached Germany in 2015, more than one third were younger than 18 years. Most of them came from Syria, Iraq and Afghanistan [1].

Tuberculosis (TB) incidence rates in Germany had seen little change for several years until 2013, when numbers of TB cases started to rise. In 2015, an increase in incidence of 29.4% compared with the previous year was recorded [2]. Among the 5,865 cases notified there were 196 children up to 15 years of age. Incidence rates of TB are linked to migration with almost three quarters of TB patients in 2016 in Germany being foreign-born [3]. In foreign-born children the incidence of TB is 37 times higher than in children born in Germany (21.4 vs 0.6/100,000 children).

Asylum seekers have a higher risk of exposure to TB, both in their countries of origin and during migration. Furthermore, an increased risk for disease progression of latent tuberculosis infection (LTBI) due to physical and psychosocial stress factors has been observed [4].

In Germany, asylum seekers aged 15 years and older entering reception centres require a medical certificate stating the absence of any signs of potentially infectious pulmonary TB based on chest X-ray findings [5]. For children and adolescents up to the age of 15 years, an immunodiagnostic screening for TB, either by tuberculin skin test (TST) or interferon gamma release assay (IGRA), is recommended [6]. Immunodiagnostic tests can identify children with LTBI before they develop active TB [7]. In these children, preventive treatment significantly reduces the risk of progression and therefore further spread of the disease and further costs associated with severe TB, especially in young children [8,9].

The approach to screen all asylum seekers for LTBI and active TB would entail considerable pressure on healthcare resources. Responding to the European refugee crisis in 2015, many countries have therefore concentrated exclusively on screening for active TB [10] and so far, there is no uniform screening procedure for asylum seekers in Europe [10].

The aim of this study was to investigate the prevalence of TB infection in refugees up to 15 years of age in two geographic regions in Germany by immunological screening. The screening was carried out as part of the initial medical screening of asylum seekers at the reception centres (‘Erstaufnahmeeinrichtung’) upon arrival in Germany.

## Methods

Between September 2015 and November 2016, children and adolescents aged from 3 months up to 15 years, were screened for TB infection using TST (2 IU PPD RT 23, Statens Serum Institute, Copenhagen, Denmark) and/or IGRA (QuantiFERON Gold in tube, Qiagen, Germantown, United States). Seven asylum seeker reception centres participated in two different German urban settings, three in Bochum and four in Hamburg. Asylum seekers in Germany usually stay in reception centres for 1 to 3 weeks before moving on to their secondary accommodation. Children who were immunised against measles, mumps and rubella (MMR) within the previous 4 weeks were excluded as live-attenuated MMR vaccines can lead to a temporary suppression of the cell-mediated immune response and result in false-negative TST results [11]. The excluded children were followed up 4 to 6 weeks later at the secondary accommodation.

A questionnaire assessed sociodemographic data including age, sex, country of origin, travel time as well as TB symptoms, TB contact (child of family member had contact with a known TB patient) and Bacille Calmette–Guérin (BCG) vaccination status.

In Hamburg, children were screened using TST and the test was considered positive if the transverse induration diameter was at least 10 mm as recommended by current guidelines [12]. In Bochum, children under the age of five were initially screened by TST, children and adolescents from 5 up to 15 years were screened by IGRA, due to a temporal shortage in TST. Screening and interpretation of TST results were the same as in Hamburg.

BCG vaccination status (mostly self-reported) and typical scars were recorded. Additionally, every child was assessed by a general medical examination. TST- or IGRA-positive children were referred to specialised children’s hospitals for further diagnostics (e.g. chest X-ray, ultrasound, IGRA, sputum or gastric aspirates) and treatment.

### Definitions

Following national guidelines, LTBI was defined as immunological evidence of infection (positive TST and/or IGRA) in absence of signs of active TB (clinical, radiological, microbiological) and active TB as evidence of TB with either microbiological and/or radiological and/or clinical signs of TB [6].

### Statistical analysis

Data were analysed using SPSS 24.0 statistics software. Descriptive statistics were calculated for all variables. Comparisons between groups were made using the t-test or the Mann–Whitney U Test. For association between categorical variables chi-squared test or Fisher`s exact test was performed. A p value less than 0,05 was considered to be statistically significant.

Consent was provided by guardians in the presence of a translator. The study was approved by local ethical committees in both Bochum and Hamburg and conducted according to current guidelines for asylum seeking children and adolescent screening [12].

## Results

A total of 1,379 asylum seeking children, aged from 3 months up to 15 years were included for evaluation in this study, 926 in Bochum and 453 in Hamburg.

We excluded 411 children from the screening: 217 had recently received MMR vaccine, 60 were screened previously in other reception centres, 80 had an acute febrile illness and 54 showed either indeterminate IGRA or were lost to follow-up (Figure). Recently vaccinated children, febrile children and those with indeterminate IGRA results received documentation regarding their current screening status and were (re-) screened at their long- term accommodation.

Of the 968 children who underwent screening the median age was 71.1 months (IQR 35–116). The majority (n=707; 73%) of the children were from the Middle East, mainly Syria (n=377; 38.9%) and Iraq (n=289; 29.9%). The third biggest group of asylum seeking children was from Afghanistan (n=217; 22.2%).

**Figure:**
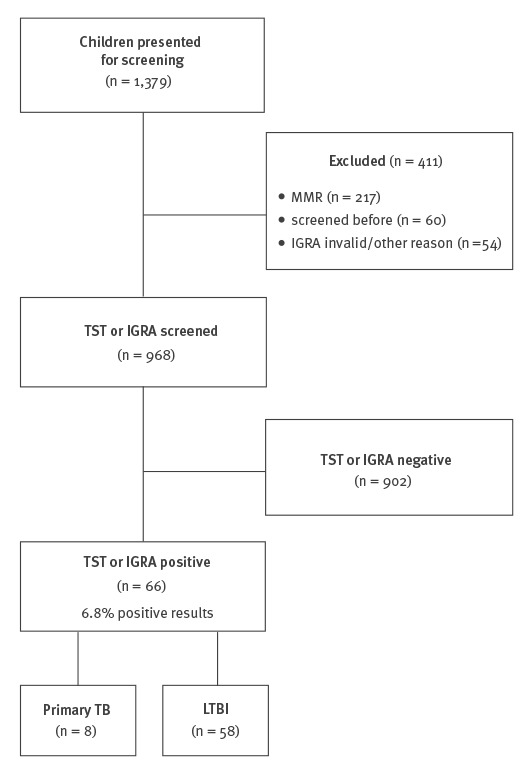
Flowchart and results of screening procedures in children and adolescents at seven asylum seeker reception centres in Bochum and Hamburg, Germany, September 2015– November 2016 (n = 1,379)

IGRA: interferon gamma release assay; LTBI: latent tuberculosis infection; MMR: measles-mumps-rubella vaccination; TB: tuberculosis; TST: tuberculin skin test.

There were 66 children and adolescents with positive TST or IGRA results. Median age was 80.5 months (range: 6–168; IQR 42.7–141.5); 38 (57.6%) were boys. Of all infected children 24 (36.3%) were from Syria, 20 (30.3%) from Iraq and 19 (28.8%) from Afghanistan. In total, 31 children (47.7%) were under the age of 6 years at the time of screening. All 66 children and adolescents with positive TST or IGRA results were referred to a paediatric hospital for further diagnostics and therapy; 58 children (32 boys, 26 girls) were diagnosed with LTBI and treated with isoniazid and rifampicin for 3 months [13]. Eight children (6 boys, 2 girls) were diagnosed with active TB by chest X-ray and/or microbiological investigations and treated according to national recommendations [6]. Among these eight there were one sputum smear-positive adolescent and two children with PCR and microbiological cultures positive for *M. tuberculosis* complex.

Sociodemographic data and screening results are summed up in the Table.

**Table:** Sociodemographic data of screened children by screening results in seven asylum seeker reception centres in Bochum and Hamburg, Germany, September 2015– November 2016

	Total		LTBI		Active TB		LTBI and active TB	
	N	%	n	%	n	%	n	%
**Number of children**	968	100	58	6	8	0.8	66	6.8
**Age** (median months)	71.1	NA	99	NA	55.5	NA	80.5	NA
**Male**	516	53.3	32	55.2	6	75	38	57.6
**Country of origin**								
Syria	377	38.9	21	36.2	3	37.5	24	36.4
Iraq	289	29.9	17	29.3	3	37.5	20	30.3
Afghanistan	217	22.4	17	29.3	2	25	19	28.8
Other^a^	85	8.8	3	5.2	0	0	3	4.5
**BCG vaccinated**	558	57.6	34	58.6	5	62.5	39	39.1
**Travel time** (median, in days)	21	NA	30	NA	30	NA	30	NA
**TB contact**	5	99.5	0	0	0	0	0	0
**TB symptoms**	69	7.1	2	3	1	12.5	1	0.02

BCG: Bacille Calmette-Guérin; LTBI: latent tuberculosis infection; NA: not applicable; TB: tuberculosis.

^a^ Other: Armenia (n= 1); Azerbaijan (n= 1); Georgia (n=5); Iran (n=41); Nigeria (n=11); Albania/Kosovo* and Serbia (n=14); Somalia (n=5); stateless (n=7).

*This designation is without prejudice to positions on status, and is in line with UNSCR 1244/1999 and the ICJ Opinion on the Kosovo declaration of independence.

### Children with latent tuberculosis

Children with LTBI were older (median age 80.5 vs 71.1 months, p=0,057) than children without TB infection. The majority was from Syria (n=21; 36.2%), followed by Iraq (n=17; 29.3%) and Afghanistan (n = 17; 29.3%). These percentages correlate well with the overall distribution of asylum seekers’ countries of origin (Table).

The prevalence of LTBI differed only slightly between children from high and low incidence countries: Afghanistan (17/217; 7.8%) vs Syria (21/377; 5.6%; p= 0,27 ) and Iraq (17/289; 5.9%; p= 0,44) . Although asylum seeking children with LTBI were older than the ones without LTBI, those under the age of 6 years were at higher, although not statistically significant risk of progression to active TB (19% vs 2% respectively; p=0,074). There were no significant differences regarding sex, travel time, symptoms or BCG vaccination status between children with and without TB infection.

### Children with active tuberculosis

The eight children with active TB were younger than the total of the screened children and those with LTBI (median 55.5 months vs 71.1 vs 80.5 months, p= 0,1); six of them were male. Of the eight children, three were from Syria and three from Iraq. The other two were from Afghanistan. There were no significant differences in travel time and BCG vaccination in comparison to the total cohort. Seven children did not report any symptoms suggestive for TB (prolonged cough, fever, night sweats). None of them reported contact with a TB patient.

## Discussion

Universal screening for TB detected 6.8 % children with LTBI and active TB in our study population of asylum seeking children up to the age of 15 years. About half of the children with LTBI were younger than 6 years and therefore at high risk of progression to active and especially disseminated TB if not treated promptly [14]. We also identified eight children in this age group who already progressed to active TB. In a large cohort study from Amsterdam, the risk of developing active TB was slightly lower for infected school age children but still reported to be up to 19.1% [15]. We identified two cases of active TB in this age group. Thus, our findings stress the importance of including children and young adolescents in national screening strategies.

Even though the overall risk of TB transmission by children is lower than by adults [14], there are reported cases in which school-aged children with active TB infected up to 39% of their contacts [16]. Considering the situation in crowded asylum seeker accommodations, the risk of transmission might be even higher [17]. The risk of developing active TB when infected has been shown to be higher in adult asylum seekers in comparison to the population in their countries of origin [18,19]. It remains unclear whether this is the case in children as well. An immunological screening of asylum seeking children of all age groups could potentially be helpful in preventing cases of infectious TB and in minimising the risk of progression to active and possibly disseminated disease in young children.

One approach to screening refugee children is to screen only children from countries with high TB incidence (above 100 cases/100,000 population). This approach does not seem ideal for different reasons [20]. Our data show that LTBI and active TB are not restricted to children from high incidence countries like Afghanistan (189 cases/100,000 population) [21]. Syrian children, the largest group of our study population, had similar percentages of TB infection as children from Afghanistan. The International Organization for Migration (IOM) and the World Health Organization (WHO) documented rising TB incidence in Syria since 2013 [4]. For 2015, the official TB incidence is reported to be 20 cases per 100.000 inhabitants by the WHO, but it is doubtful whether this figure reflects the actual numbers as precise recording in times of war and civil unrest is known to be difficult [4].

Another reason for the increased numbers of TB infection in Syrian and Iraqi asylum seeking children might be related to travel routes and travel time to Germany. Of the 44 children from Syria and Iraq who had positive screening results, 13 spent a month or more in refugee camps along the Balkan route or before crossing the Mediterranean Sea. Very often medical support and nutrition during migration are inadequate and refugees from high and low TB incidence countries live together in crowded accommodations.

Symptom-based screening of refugees has been standard practice in many countries [10]. However, sensitivity and specificity of symptom-based screening is poor [22-24] and does not represent an effective screening approach, especially in young children [24]. In our study only one child with active TB displayed suggestive symptoms, while seven were detected by immunological screening and subsequent investigations.

Responding to the enormous increase in asylum seekers in 2015, many local authorities in Germany and other Western European countries concentrated on screening direct contacts of infectious TB patients [10,12]. Our data shows that contacts to TB cases were either not remembered or not stated by the children or their guardians, maybe due to fear of stigma connected with TB.

In our experience, universal TB screening in asylum seeking children is both effective and feasible. However, documentation of screening results and assurance of treatment completion in infected and diseased children remains crucial.

In addition to recording the screening result in the national vaccination card , asylum seeking children in the two urban areas involved in this study were supposed to receive a refugee health document (North Rhine-Westphalia health card (NRW Gesundheitskarte)); Hamburger health booklet (Hamburger Gesundheitsheft) in order to collate existing data and prevent redundant examinations. Despite the large overall amount of data that are collected in the process of providing healthcare to asylum seekers, there are neither federal standards for documentation, nor is there a standard set of collected health items in Germany [25,26]. In view of national and international mobility of asylum seekers, an innovative European solution of health information management would be desirable for the future.

Preventive therapy of LTBI in children is well tolerated and prevents progression to active TB in more than 90% [13]. Therefore, reduction of cases of active TB seems feasible in asylum seeking children. In order to assure treatment completion, different challenges have to be tackled in this hard-to-reach population. The stigma of being diagnosed with TB has to be addressed and resolved. Easy access to healthcare and increased tuberculosis awareness are vital [27]. In addition, directly observed therapy (DOTS) in asylum seeker reception centres and subsequent housing facilities could improve success rates.

Cost-effectiveness of TB screening was not investigated in our study. In a recent review in adults, LTBI screening in asylum seekers was described as cost effective [28]. None of the included studies, however, evaluated the subgroup of children.

An ideal approach would be to assess the overall exposure risk and perform baseline TB screening for all asylum seekers – both children and adults – upon arrival and pursue follow-ups over the next 2 years.

The ‘End TB Strategy’, adopted by WHO Member States in 2014, identifies TB in hard-to-reach populations – such as asylum seekers– as an important public health challenge for low-incidence countries like Germany and other Western European countries and recommends the implementation of comprehensive social and healthcare interventions [29].

Even though we screened a comparably high number of children, there are various study limitations that need to be taken into consideration. First our cohort is likely not to be representative for the general population of asylum seekers that reached Germany during the time of our investigation. It rather presents a snapshot at the moment of data collection, when the total numbers of asylum seekers, even in single reception centres, were not reliably documented. The distribution of asylum seekers within Germany was very heterogeneous for example regarding the country of origin and therefore a nationwide study would have been desirable. Another problematic issue was the high mobility of the asylum seekers. Anecdotal evidence showed that the latter were frequently registered twice at different centres and moved freely between them. Therefore, either double or incomplete registration could have caused a bias regarding epidemiological data, follow-up investigations and treatment [25,26].

## Conclusion

In conclusion, our data supports the implementation of universal screening for LTBI and active TB in asylum seeking children of all ages and from both high and low TB incidence countries using immunological tests such as TST or IGRAs in order to prevent future active TB cases and further spread of the disease. Careful documentation of screening results and completion of preventive therapy should be ensured to guarantee the success of this screening approach.
